# Trehalose 6-phosphate signal is closely related to sorbitol in apple (*Malus domestica* Borkh. cv. Gala)

**DOI:** 10.1242/bio.022301

**Published:** 2017-01-09

**Authors:** Wen Zhang, John E. Lunn, Regina Feil, Yufei Wang, Jingjing Zhao, Hongxia Tao, Yanping Guo, Zhengyang Zhao

**Affiliations:** 1 College of Horticulture, Northwest A&F University, Yangling, Shaanxi 712100, China; 2 Max Planck Institute of Molecular Plant Physiology, Metabolic Networks Group, Am Mühlenberg 1, Potsdam-Golm D-14476, Germany; 3 Key Laboratory of Horticulture Plant Biology and Germplasm Innovation in Northwest China, Ministry of Agriculture, Northwest A&F University, Yangling, Shaanxi 712100, China; 4 Shaanxi Engineering Research Center for Apple, Northwest A&F University, Yangling, Shaanxi 712100, China

**Keywords:** Apple, Signaling, Sorbitol, Sugar, Tre6P

## Abstract

Trehalose-6-phosphate (Tre6P) is a precursor of trehalose, which is widespread in nature and greatly influences plant growth and development. Tre6P acts as a signal of carbon availability in many plants, but little is known about the function of Tre6P in rosaceous plants, which have specific sorbitol biosynthesis and transportation pathways. In the present study, Tre6P levels and Sorbitol:Tre6P ratios were analyzed in apple (*Malus domestica*, Borkh. cv. Gala). Tre6P levels were positively correlated with sorbitol content but negatively correlated with sucrose, glucose, and fructose content in developing fruit. However, under sorbitol-limited conditions, Tre6P levels were positively correlated with both sorbitol and sucrose. In the presence of different exogenous sugar supply, Tre6P levels increased corresponding with sorbitol, but this was not the case with sucrose. In addition, Tre6P content and sorbitol:Tre6P ratios were more highly correlated with ADP-glucose levels under sorbitol-limited conditions and fruit development stages, respectively. These results suggest that Tre6P is more closely related to sorbitol than other soluble sugars and has an important role in influencing carbon metabolism in apple.

## Abstract (precis)

**Summary:** Metabolite analysis of the Tre6P pathway in apple fruit, *Malus domestica*, reveals that Tre6P levels were highly and positively correlated with sorbitol content, but not with sucrose content.

## INTRODUCTION

Sugar biosynthesis is one of the most crucial metabolic processes in plants (Shangguan et al., 2014). Sugars act as metabolic resources and regulators of several processes associated with embryo development (Borisjuk et al., 2004; Hills, 2004), seedling development (Dekkers et al., 2004; Price et al., 2003; To et al., 2003), and maturity (Fujikake et al., 2003; Hanson et al., 2001). The pathways of sorbitol biosynthesis and transportation are specific in rosaceous plants, such as apple and pear trees. Sorbitol accounts for approximately 80% of the total soluble carbohydrates in apple leaves, whereas sucrose accounts for only 10% (Loescher et al., 1982). A growing apple fruit is a sink organ that accumulates fructose and sucrose. Starch is also synthesized but subsequently degraded in later stages of fruit development (Hulme, 1958). To understand the carbon economy of fruit production, it is necessary to determine the regulatory mechanisms involved in sugar and starch synthesis.

Trehalose-6-phosphate (Tre6P) is an intermediate product of trehalose biosynthesis. Tre6P is a product of the reaction between UDP-glucose (UDPG) and glucose-6-phosphate (Glc6P) (Cabib and Leloir, 1958), which is catalyzed by trehalose-6-phosphate synthase (TPS; EC 2.4.1.15). Tre6P is further metabolized to trehalose by trehalose-6-phosphate phosphatase (TPP; EC 3.1.3.12) (O'Hara et al., 2013), which is eventually hydrolyzed by trehalase into glucose (Elbein et al., 2003). Martins et al. (2013) found that Tre6P is predominantly located in the cytosol (71% to 72%), with 16% to 22% being located in chloroplasts and 7% to 11% in the vacuoles of *Arabidopsis thaliana* rosettes.

Numerous studies have confirmed the correlation between Tre6P and sucrose throughout the diurnal cycle, during leaf senescence, in response to cold and altered nitrogen (N) availability in *A. thaliana* (Veyres et al., 2008; Wingler et al., 2012; Nunes et al., 2013; Sulpice et al., 2014), and in developing potato tubers (Debast et al., 2011) and wheat grains (Martínez-Barajas et al., 2011). Tre6P promotes growth when sucrose supply is high (Schluepmann et al., 2004; Lunn et al., 2006), and growth is inhibited when Tre6P is not increased in balance with carbon availability (Yadav et al., 2014). Thus, with a resupply of sucrose, Tre6P content is coordinately and rapidly elevated, peaking at over 25 times higher than that in carbon (C)-starved seedlings (Lunn et al., 2006). The overexpression of *TPS* and *TPP* genes in *A. thaliana* indicate that Tre6P, rather than trehalose (Schluepmann et al., 2003), is responsible for the development of leaves and branched inflorescences (Wahl et al., 2013). Therefore, Tre6P acts as a signal for high carbon availability in the form of sucrose.

Starch regulatory pathways are controlled through a complex network. Kolbe et al. (2005) reported that Tre6P acts as an intermediary, increasing the rate of starch synthesis via the redox activation of AGPase. Lunn et al. (2006) also observed parallel changes in sucrose, Tre6P, and the redox status of AGPase in *Arabidopsis* rosettes. However, Martins et al. (2013) reported that Tre6P did not promote the activation of AGPase in TPS mutant *Arabidopsis* leaves and that AGPase has little influence on starch synthesis. Martins et al. (2013) further proposed that the rate of starch breakdown is synergistically regulated through the circadian clock and Tre6P, with the clock-dependent regulation preventing the premature exhaustion of starch before dawn.

Trehalose-6-phosphate has no significant inhibitory effects on the hexokinase activities of spinach (Wiese et al., 1999), *Arabidopsis* (Eastmond et al., 2002), or tomato (Kandel-Kfir et al., 2006). Yadav et al. (2014) also found that Tre6P indirectly responds to glucose or fructose but is directly influenced by sucrose. However, it is not yet clear whether Tre6P responds specifically to sucrose or is a more general signal of sugar availability (Yadav et al., 2014). In the present study, correlations among sucrose, hexose sugars, starch and Tre6P content were analyzed. Apple (*Malus domestica*) with or without sorbitol limitations were used to investigate whether Tre6P is highly correlated to sorbitol. In addition, correlations among sorbitol:Tre6P ratios, soluble sugars, and starch in apple fruit were analyzed.

## RESULTS

### Growth parameters of apple fruit

**Fig. 1.**
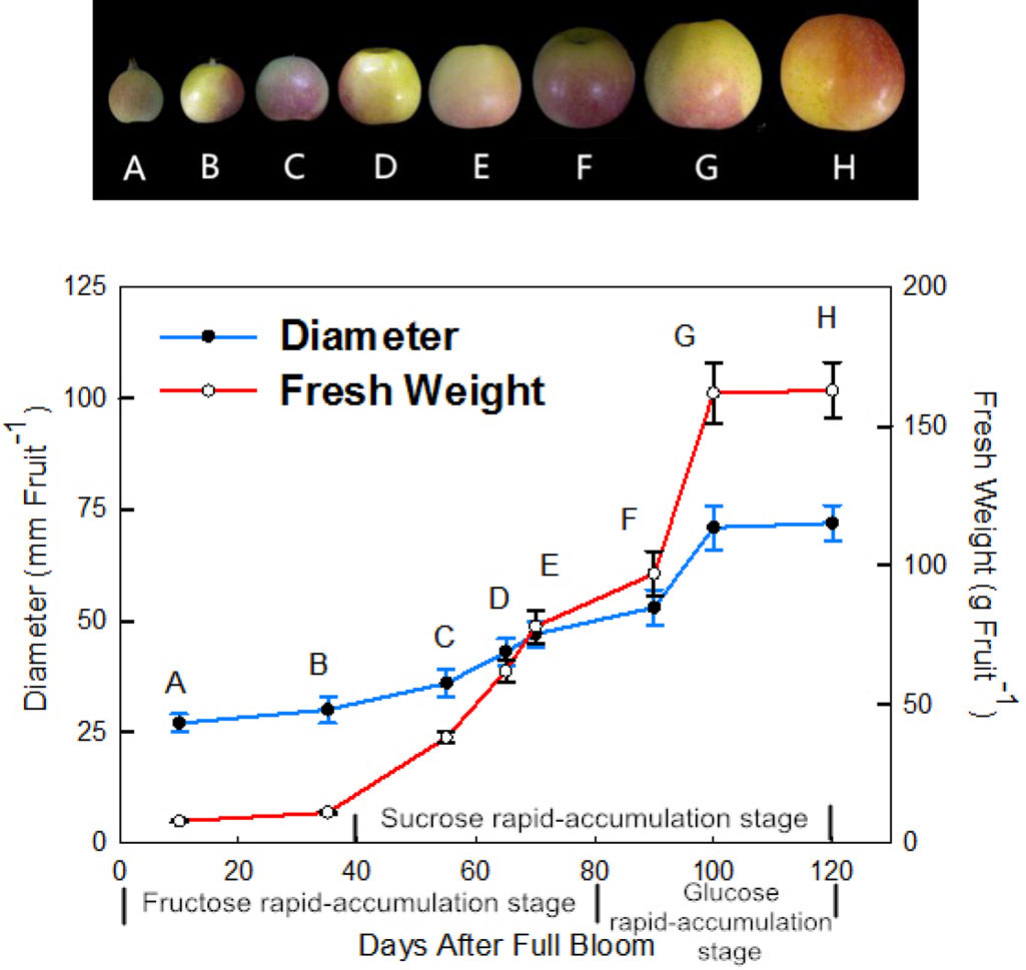
**Typical fruit phenotypes and growth parameters of the developing ‘Gala’ apple.** A to H represent different growth phenotypes: A, 10 DAFB; B, 35 DAFB; C, 55 DAFB; D, 65 DAFB; E, 70 DAFB; F, 90 DAFB; G, 100 DAFB; H, 125 DAFB. Each point is mean±s.e.m. (*n*=10).

A profile of apple fruit growth was established by measuring the changes in single fruit mass and transverse diameter during fruit development (Fig. 1). Apple fruit development took approximately 120 days from full blooming flowers to fruit ripeness. The fruit growth followed the slower-fast-slower rule during this time in which the fruit grew slowly in the first 35 days, and then rapidly expanded in growth from 35 days after full bloom (DAFB) to 105 DAFB, which is called the rapid growth period. Subsequently, from 105 to 120 DAFB the second slow growth period of fruit was observed.

### Metabolic profiling during fruit development

**Fig. 2.**
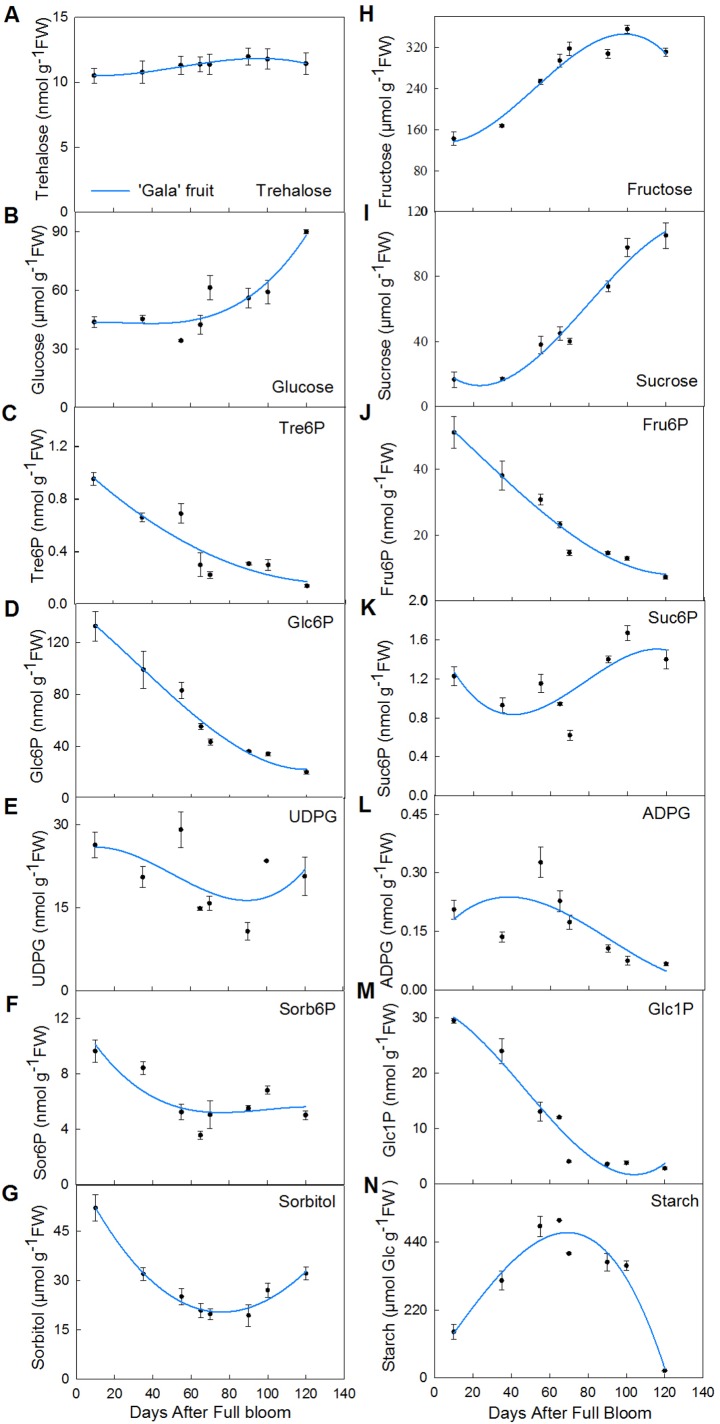
**Primary metabolite levels in developing apple fruit.** A to N represent different primary metabolites: A, trehalose; B, glucose; C, trehalose 6-phosphate; D, glucose 6-phosphate; E, UDP-glucose; F, sorbitol 6-phosphate; G, sorbitol; H, fructose; I, sucrose; G, Fru6P; K, sucrose 6-phosphate; L, ADP-glucose; M, glucose 1-phosphate; N, starch. Each values are means±s.e.m. (*n*=3).

Soluble sugars, such as fructose and sucrose, were the main sugars identified during apple fruit development (Shangguan et al., 2014). Although the speed of fructose accumulation was slow from 80 to 120 DAFB, fructose content was the greatest among all soluble sugars examined (Figs 1 and 2). As shown in Fig. 2G, the sorbitol levels initially and rapidly decreased during development and subsequently accumulated gradually. The sucrose and glucose content did not significantly accumulate until 40 and 80 DAFB, respectively (Fig. 1, Fig. 2B,I). The starch content increased from the time of full bloom, peaking at 70 DAFB, and rapidly decreased to a low level thereafter (Fig. 2N). Therefore, these results, consistent with Li et al. (2012), indicated that mature apple fruit had lower sorbitol and starch contents but higher fructose, glucose and sucrose concentrations (Fig. 2).

### Content of Tre6P in developing fruit

Trehalose-6-phosphate, as a sugar signal in plants (Zhang et al., 2009), was assayed in developing apple fruit (Fig. 2C). The Tre6P values decreased, ranging from 0.95 to 0.14 nmol g^−1^ fruit weight (FW), during continuous growth, markedly decreased prior to 70 DAFB, and subsequently showed a slight decrease thereafter. Thus, young fruit contained more Tre6P content than mature fruit. The Tre6P content of apple, particularly younger fruit, were higher than those of the *A. thaliana* leaf and shoot apical meristem (Wahl et al., 2013; Yadav et al., 2014) but lower than those of cucumber peduncles (Zhang et al., 2015) and maize spikelets (Nuccio et al., 2015).

### The independence of Tre6P

Trehalose levels were stable from 20 DAFB to 70 DAFB and slightly increased after 70 DAFB in developing fruit (Fig. 2A). Trehalose-6-phosphate, as the precursor of trehalose (O'Hara et al., 2013), was not feedback regulated through trehalose levels (Fig. 2A,C). Thus, Tre6P did not directly respond to the changes of trehalose content in developing fruit. The embryo-lethal *A. thaliana tps1* mutants were rescued through the expression of *E. coli TPS*, but not by the exogenous supply of trehalose (Schluepmann et al., 2003). Taken together, these results obtained in apple and *A. thaliana* suggest that Tre6P was independent from trehalose content.

### Changes in Tre6P precursors with fruit development

The contents of Glc6P and UDPG, as the precursors of Tre6P, changed with fruit growth (Fig. 2D,E). The trends of Glc6P content were highly similar to those of the Tre6P levels, while the trends of UDPG levels were not obviously decreased (Fig. 2D,E).

Glucose more readily enters the hexose phosphate pool and transforms into starch during fruit development (Beruter et al., 1997); thus, Glc6P is derived as an intermediate product during the conversion of glucose into starch in apple development (Shangguan et al., 2014). In the present study, the glucose content did not significantly accumulate until 70 DAFB (Fig. 2B), during which starch levels rapidly increased, peaking at 70 DAFB (Fig. 2N). As an intermediate product between glucose and starch, Glc6P sharply decreased with glucose degradation and starch synthesis from 20 DAFB to 70 DAFB (Fig. 2B,D,N). Starch content decreased after 70 DAFB during which Glc6P levels declined slowly and glucose content accumulated. Glucose, starch, and Glc6P content are coordinated with fruit development.

UDPG could be synthesized from sucrose and Glc1P (Li et al., 2012; Shangguan et al., 2014). The starch content favored synthesis prior to 70 DAFB, during which Glc1P markedly decreased (Fig. 2I,M,N). UDPG slightly decreased with decreasing Glc1P levels, and sucrose sharply increased from 10 to 70 DAFB (Fig. 2E,I,M). When the starch contents sharply decreased, Glc1P levels were not markedly changed (Fig. 2M,N). Although the trends of Glc1P were steady, the UDPG content was accumulated with increasing sucrose (Fig. 2I,M,N). UDPG appears to play a role in sucrose and starch biosynthesis pathways.

Glucose is derived from sucrose and sorbitol (Beruter et al., 1997), and sucrose content is derived from photosynthetic transportation and re-synthesis through intermediates, such as Fru6P and Suc6P (Shangguan et al., 2014) in apple. Hence, Glc6P and UDPG levels were not only influenced by glucose, sucrose, and starch content but also by sorbitol, Fru6P, Suc6P and other metabolites in developing apple fruit. Thus, Tre6P levels had a close relationship with soluble sugars and starch in fruit development.

### Correlation between Tre6P and primary metabolites during growth

To better understand the mechanisms of Tre6P in developing fruit, we performed a correlation analysis as shown in Table 1. The positive correlation between Tre6P and sorbitol was significant (*P*=0.049), while Tre6P was negatively correlated with sucrose (*P*=0.028) and fructose (*P*=0.003). Although the correlation between Tre6P and glucose was not statistically significant, the correlation of Tre6P with glucose (*P*=0.056) was close to the 0.05 significance level (*P* value). However, Tre6P showed little correlation with ADPG (*P*=0.167) or starch (*P*=0.917). There were also significant correlations among sorbitol, sucrose, fructose and glucose. The soluble sugars in apple fruit were significantly correlated with the Tre6P levels, but it is difficult to determine which sugar is directly correlated with Tre6P.

**Table 1.**
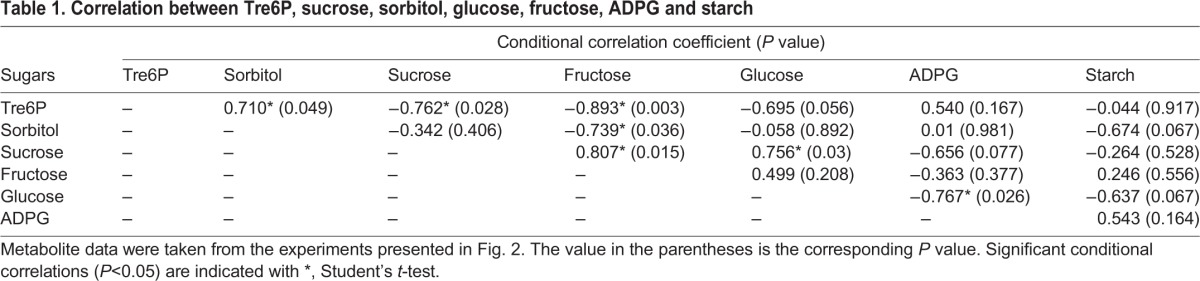
**Correlation between Tre6P, sucrose, sorbitol, glucose, fructose, ADPG and starch**

### Responses of Tre6P, sucrose and hexose sugars to sorbitol limitation

**Fig. 3.**
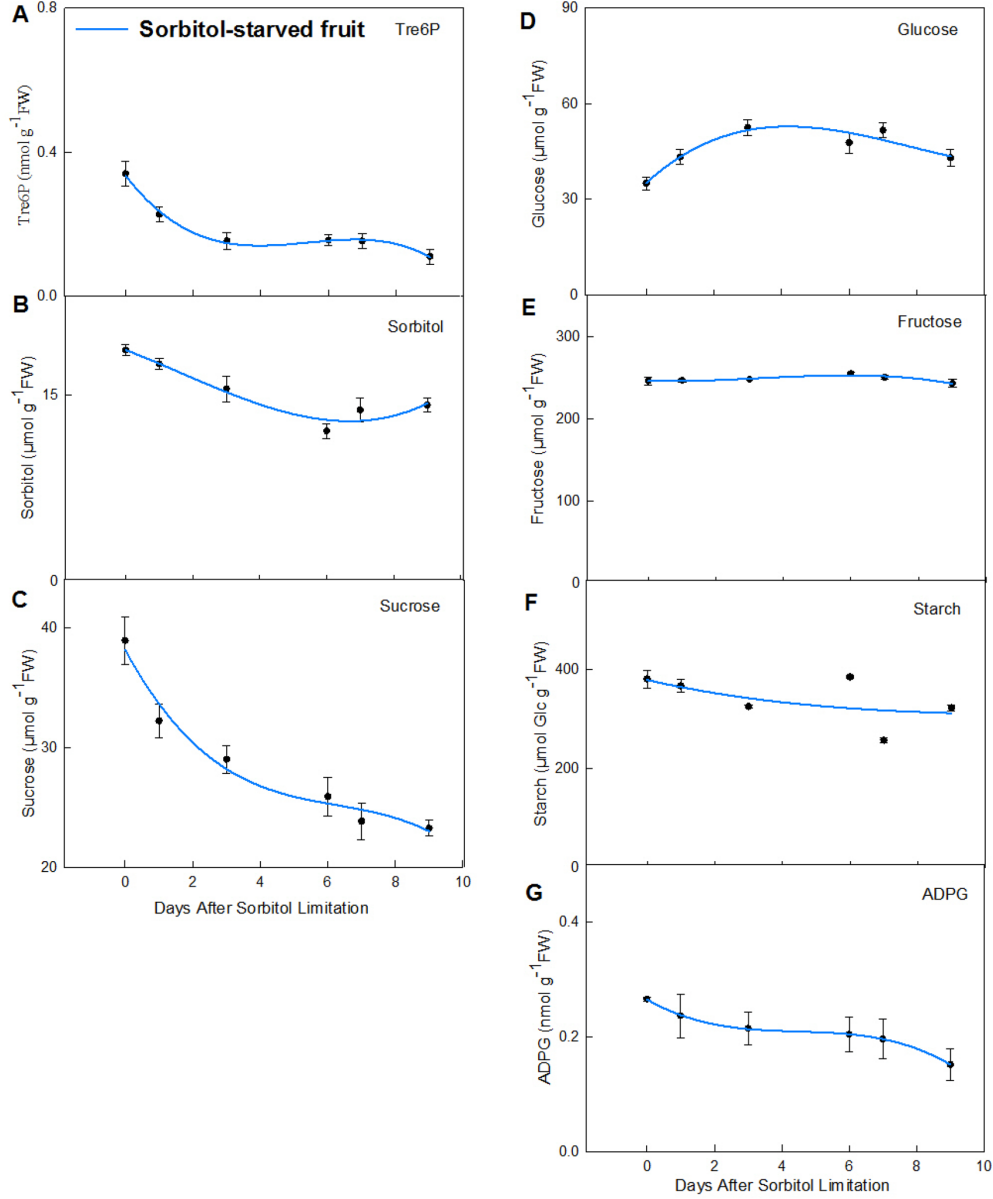
**Changes of Tre6P, sucrose and hexose sugars with sorbitol starvation.** A to N represent different primary metabolites: A, trehalose 6-phosphate; B, sorbitol; C, sucrose; D, glucose; E, fructose; F, starch; G, ADP-glucose. Each point is mean±s.e.m. (*n*=3).

To better understand the nexus between soluble sugars and Tre6P, apple fruit was subjected to sorbitol limitation through leaf thinning. The results showed that decreasing levels of sorbitol were accompanied by fluctuations in sugar and starch content, including Tre6P (Fig. 3). Tre6P levels were rapidly reduced from 0 to 3 starvation days and thereafter slightly decreased. Sorbitol and sucrose content both rapidly decreased during the first 6 starvation days. Glucose content was first increased and then reduced while fructose, starch, and ADPG levels all had small fluctuations due to sorbitol limitation. Thus, Tre6P, soluble sugars and starches were all obviously influenced by sorbitol levels.

Correlations were analyzed among Tre6P, sugars, and starches in sorbitol-starved fruit (Table 2). In addition to sorbitol (*P*=0.044), Tre6P was also positively and significantly correlated with sucrose (*P*=0.002) and ADPG (*P*=0.011). Instead of a sucrose correlation with sorbitol (*P*=0.406) in developing fruit (Table 1), there was a significant correlation (*P*=0.02) in sorbitol-starved fruit (Table 2). Moreover, unlike the relationship among ADPG, sucrose, and glucose in developing fruit (Table 1), ADPG levels were more closely associated with sucrose (*P*=0.008) than with glucose (*P*=0.377) under sorbitol-limited conditions (Table 2).

**Table 2.**
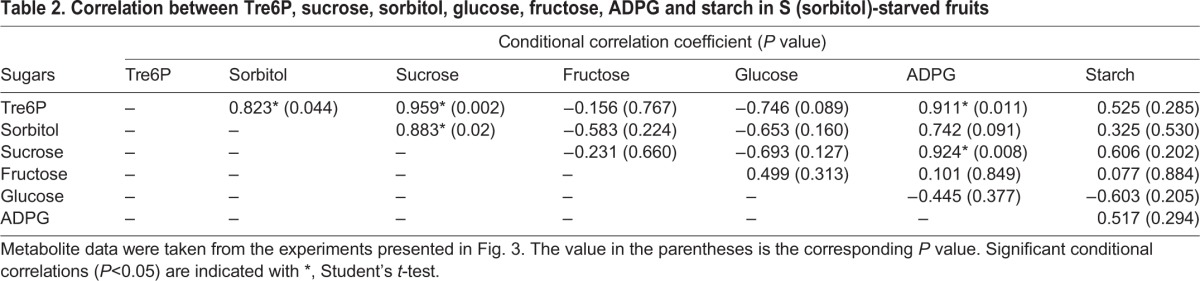
**Correlation between Tre6P, sucrose, sorbitol, glucose, fructose, ADPG and starch in S (sorbitol)-starved fruits**

### Supplementation of sugars in sorbitol-starved fruit over different time periods

Sorbitol-starved fruit were supplied with exogenous sugars for 2 or 4 days to explore the relationships among sugars (Table 3). When one sugar was supplied, the levels of the other sugars and Tre6P were all more or less changed. With glucose or sucrose addition for 2 days, only Tre6P, sucrose, and sorbitol were markedly increased. However, with fructose addition for 2 days, the Tre6P content paralleled steady sorbitol trends and did not show an increase with increasing sucrose levels. With the addition of sorbitol or sucrose for 4 days, Tre6P and other sugar content, but not glucose content, were significantly elevated. When fructose was added for 4 days, the other sugars were also all significantly increased, with the exception of a significant decrease of glucose. However, when glucose was added for 4 days, all other soluble sugars and Tre6P levels were elevated. Thus, Tre6P was more sensitive to glucose and sucrose than to sorbitol, but Tre6P levels only paralleled sorbitol content under all sugar supplementation conditions.

**Table 3.**
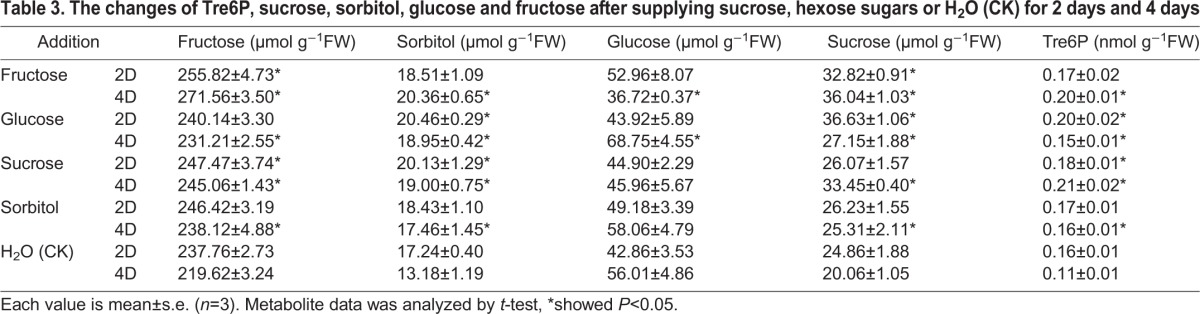
**The changes of Tre6P, sucrose, sorbitol, glucose and fructose after supplying sucrose, hexose sugars or H_2_O (CK) for 2 days and 4 days**

### The sorbitol:Tre6P ratio and starch content

As shown in Table 1, neither Tre6P nor sorbitol were significantly correlated with ADPG or starch; however, the sorbitol:Tre6P ratios were positively correlated with starch (*P*=0.056) (Table 4). In addition, the sorbitol:Tre6P values in growing fruit were significantly correlated with glucose (*P*=0.000) and sucrose (*P*=0.045). Although the glucose content was significantly correlated with ADPG (*P*=0.026) (Table 1), the sorbitol:Tre6P value had no obvious correlation with ADPG (*P*=0.120) (Table 4). The ratio of sorbitol to Tre6P is not only highly connected to starch content, but also to sucrose and glucose contents with fruit development.

**Table 4.**
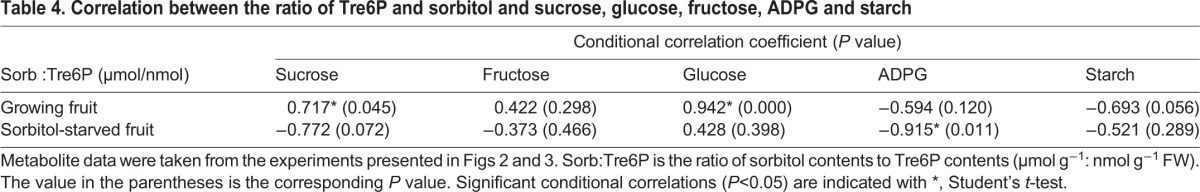
**Correlation between the ratio of Tre6P and sorbitol and sucrose, glucose, fructose, ADPG and starch**

## DISCUSSION

### Changes in soluble sugars and Tre6P in developing fruit

Changes in carbohydrate metabolism during fruit development play an important role in shaping the final sugar composition and content of apple fruit (Dai et al., 2013). Sorbitol acts as the main transportation agent in rosaceous plants (Shangguan et al., 2014) and is used to synthesize other sugars, such as fructose, sucrose, and glucose in apple fruit. Sorbitol initially decreased with fruit growth from 10 to 70 DAFB and subsequently only slightly accumulated from 18.35 to 29.34 μmol g^−1^ FW (Fig. 1, Fig. 2G). Fructose was the most highly accumulated sugar (Li et al., 2012), predominantly accumulating from 10 to 80 DAFB during fruit development (Fig. 1, Fig. 2H); sucrose content rapidly increased at 40 DAFB during fruit enlargement (Fig. 1, Fig. 2I); and glucose content rapidly increased at 80 DAFB to fruit maturity (Fig. 1, Fig. 2B). During apple fruit development, Tre6P levels rapidly decreased initially, but then the downward trend slowed down after 70 DAFB (Fig. 2C).

**Fig. 4.**
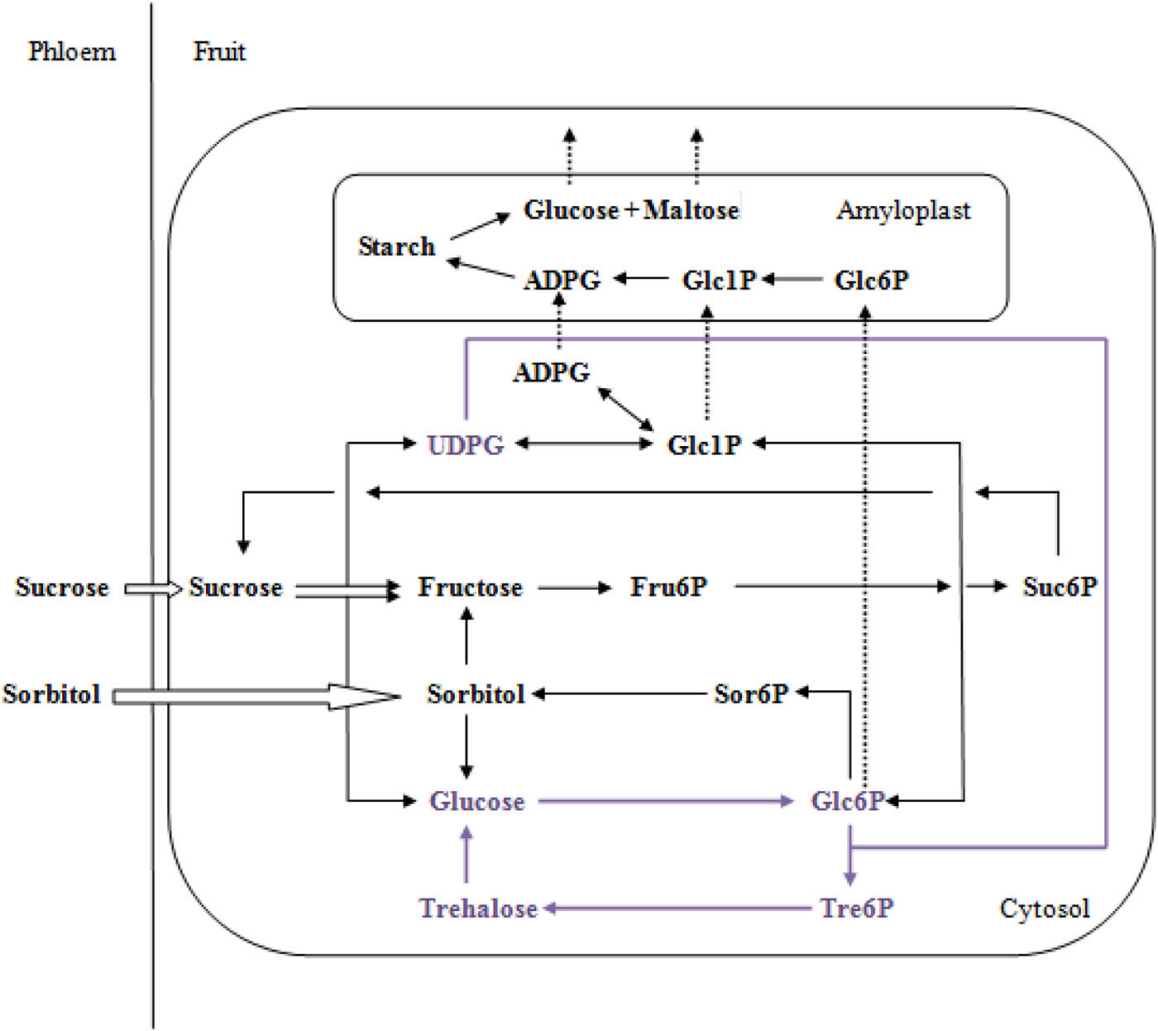
**A comprehensive and simplified model of sugar metabolism in apple fruit.** The sugar biosynthesis steps are marked with solid lines, and the trans-membrane transport are marked with dashed lines. Both sorbitol and sucrose are unloaded from phloem into the cytosol, indicated by arrow outlines. The Tre6P metabolism is marked by purple text (Debast et al., 2011; Nuccio et al., 2015; Schluepmann et al., 2003); the other metabolisms of the modal comes from apple fruit are reported in Shangguan et al. (2014).

Fruit development is a dynamic and complex process involved in the conversion and accumulation of sugars (Coombe and McCarthy, 2000). In order to better understand Tre6P and sugar metabolism, a simple biochemical model was applied to the metabolic pathways of Tre6P in apple fruit (Fig. 4). The Tre6P metabolism model was based on the results of researchers who studied potato, *Arabidopsis thaliana*, and maize (Debast et al., 2011; Nuccio et al., 2015; Schluepmann et al., 2003). In this model, both sucrose and sorbitol were transported into fruit, but sorbitol was the main component incorporated. Sorbitol or sucrose converted into Tre6P through three main steps and several smaller ones. This complex series of steps is as follows: (i) sorbitol→glucose→Glc6P; (ii) sucrose→UDPG; (iii) Glc6P+UDPG→Tre6P. The other molecules such as Fru6P, Glc1P, and trehalose also play important roles in the metabolic pathways of Tre6P.

### Tre6P levels are highly connected with sorbitol content

Tre6P acts as a signal for change in plant tissue in parallel with the sucrose content (Yadav et al., 2014). This relationship has been observed in the seedlings (Lunn et al., 2006; Nunes et al., 2013), rosettes (Veyres et al., 2008; Wingler et al., 2012; Carillo et al., 2013; Sulpice et al., 2014), and shoot apices (Wahl et al., 2013) of *A. thaliana*, developing potato tubers (Debast et al., 2011), and wheat grains (Martínez-Barajas et al., 2011). However, Tre6P was less obviously associated with sucrose during grape berry development (Dai et al., 2013). In the present study, decreased Tre6P levels were significantly and positively correlated with sorbitol levels, but negatively correlated with sucrose, glucose, and fructose contents (Table 1).

Tre6P levels were positively and strongly correlated (*P*=0.049) with sorbitol content during fruit development (Table 1). When sorbitol transportation was limited by thinning leaves, Tre6P levels were also decreased in parallel with the sorbitol content. The correlation of sorbitol-starved fruit between sorbitol and Tre6P was highly significant (*P*=0.044) (Table 2). After the addition of exogenous sorbitol to sorbitol-starved fruit, Tre6P was elevated with increasing sorbitol levels in fruit cells. Moreover, Tre6P did not respond to trehalose accumulation in apple (Fig. 2A,C), similar to the response of *A. thaliana* (Schluepmann et al., 2003).

From 20 to 90 DAFB, Tre6P levels sharply decreased, which precisely corresponded with sorbitol trends in apple. After 90 DAFB, Tre6P levels had a slight downtrend while sorbitol had a slight accumulation (Fig. 2C,G). Although there were different trends between Tre6P and sorbitol levels from 90 to 120 DAFB (Fig. 2C,G), Tre6P levels were positively and highly correlated (*P*=0.049) with sorbitol content during complete development (Table 1). In addition, sorbitol unloaded from leaves into the fruit is converted to fructose or glucose in fruit (Yamaki, 2010). Hexose benefits fruit growth by producing high osmotic pressure for active cell enlargement in developmental stages, but not in mature stages, resulting in a lot of sugar accumulation in mature fruit (Yamaki, 2010). Therefore, Tre6P was highly correlated with sorbitol during fruit development, especially from 20 to 90 DAFB when much of the sorbitol content was metabolized into hexose (Fig. 2C,G).

To investigate whether Tre6P and sorbitol had specific relationships in apple, different sugars were supplied to sorbitol-starved fruits. Hexose sugars and sucrose were more or less changed after supplying different sugars or at different time periods of development (Table 3). In *A. thaliana*, Tre6P was sensitive to sucrose content rather than the content of other sugars (Yadav et al., 2014). In apple, Tre6P levels were also more rapidly and strongly elevated after supplementation with sucrose than with sorbitol. Specifically, Tre6P was increased twofold after the addition of sucrose for 4 days, showing higher levels than those detected after the addition of other sugars. When fructose was added to sorbitol-starved fruits for 2 days, Tre6P levels were consistent with sorbitol content but not sucrose content. However, Tre6P paralleled sorbitol more than any of the other sugars irrespective of the type of sugar supplied to sorbitol-starved fruits. Thus, although Tre6P levels increased more rapidly in response to sucrose, Tre6P did not parallel the sucrose content under all conditions of sugar supplementation. Tre6P instead paralleled sorbitol content with the addition of different exogenous sugars.

### Relationships among Tre6P, sorbitol, and sucrose in apple fruit

Tre6P was positively correlated with sorbitol under both sorbitol-limited and normal growth conditions (Tables 1 and 2). Tre6P was negatively correlated with other sugars, such as sucrose, in growing fruit and Tre6P was positively correlated with sucrose levels in sorbitol-limited fruit. Similarly, Tre6P trends in developing grapes had little connection to sucrose levels (Dai et al., 2013). The changes of Tre6P levels in developing apple fruit also did not positively correlate to the sucrose content but rather to the sorbitol content (Table 1). The cells of developing apple fruit can increasingly accumulate sucrose, glucose and fructose in the vacuole, which is not the primary location of Tre6P reactions (Martins et al., 2013; Yamaki and Ino, 1992), because sorbitol and Tre6P are mainly located in cytoplasm. This might be one reason why Tre6P did not positively correlate with sucrose content in apple or grape fruit cells. On the other hand, sucrose showed no significant correlation with sorbitol during fruit development, but was highly correlated with sorbitol through sorbitol limitation. There were totally different relationships of sucrose to Tre6P and sorbitol under both normal developing conditions and sorbitol starved conditions. These results indicate that sucrose levels are regulated by a pathway not utilized under normal developing conditions. Reflecting the Tre6P main reaction origin (Martins et al., 2013), this pathway might be initiated in cytosol. Indeed, SnRK1 is activated through Tre6P levels in growing tissues (Zhang et al., 2009) and potentially affects growth and other cellular processes (Baena-González et al., 2007). Further research is needed to determine whether the SnRK1 pathway regulates sucrose content via Tre6P or sorbitol in sorbitol-starved apple fruit.

### Sorbitol coordinates with Tre6P to regulate starch content

With fruit development, starch was accumulated from approximately 10 to 70 DAFB and subsequently rapidly declined to extremely low levels, but was not totally exhausted at 120 DAFB (Fig. 2N). ADPG is an important precursor substance for starch synthesis, and the major products of starch degradation are glucose and maltose (Shangguan et al., 2014). With fruit development, ADPG content decreased and glucose content increased after 70 DAFB (Fig. 2B,L,N). In addition, starch synthesis was increasingly weaker, while starch degradation was increasingly stronger, during apple fruit development (Fig. 2B,L,N). Thus, 70 DAFB is an important time point for starch metabolism in ‘Gala’ apple.

In starch metabolism, ADPG and sucrose influence starch synthesis and degradation. Kolbe et al. (2005) found that the Tre6P signal influenced starch contents by regulating ADPG content in potato tubers. However, Martins et al. (2013) indicated that the rate of starch degradation in *Arabidopsis* leaves is regulated by the circadian clock instead of AGPase via sucrose and Tre6P. In plants, it is not clear whether AGPase or the circadian clock is involved in the regulation of starch contents through the Tre6P signal. Yadav et al. (2014) reported that the Tre6P:sucrose ratio is a critical parameter for *A. thaliana* development, helping to form part of a homeostatic mechanism that maintains sucrose levels within a specific range to regulate plant development. In this study, neither sorbitol nor Tre6P was strongly correlated with starch content (Table 1) in developing fruit; however, sorbitol:Tre6P values were closely linked to starch content (*P*=0.056) and were highly and significantly correlated with glucose (*P*=0.000) during fruit development (Table 4). In sorbitol-starved fruits, the ratios of Tre6P to sucrose were not significantly correlated to starch content (*P*=0.289), but were more closely related to ADPG (*P*=0.056) (Table 4). Therefore, there might also be two regulation pathways that influence starch accumulation in apple fruit under normal and sorbitol-starved development. The connection between sorbitol:Tre6P ratios with starch and ADPG contents were observed in apple. Thus, the mechanisms driving sorbitol:Tre6P ratios as related to starch content needs further study under different growth conditions.

In conclusion, sorbitol content instead of sucrose was highly and positively correlated with Tre6P levels with or without sorbitol limitation in apple. Moreover, Tre6P levels synchronously changed only with sorbitol content under all conditions of sugar supplementation. To obtain a complete understanding of Tre6P function, future studies should investigate whether the Tre6P-sorbitol nexus relates to apple leaves or other rosaceous plants, and further characterize the starch metabolic pathways that utilize the Tre6P signal.

## MATERIALS AND METHODS

### Plant materials

Biochemicals were obtained from Sigma-Aldrich Shanghai Trading Co Ltd (Shanghai, China) unless otherwise indicated. [6,6-2H]Tre6P was enzymatically synthesized from [6,6-2H]d-glucose (Lunn et al., 2006).

Five-year-old apple fruit trees (*Malus domestica* Borkh. cv. Gala) were grown in an orchard at Northwest A&F University, Shaanxi, China (34°20 N, 108°24 E). The 40 trees were grown in 45 cm×50 cm plastic containers in 20 kg of medium composed of top soil:turf soil at a 1:1 (v/v) ratio under natural conditions. The source:sink ratio of the apple trees were approximately 35:1 (3 fruit per m^2^ leaf area), and the trees were watered with 4–6 liters every one or two days using a trickle irrigation system. To protect the plants from disease and insects, fungicides and pesticides were sprayed at regular intervals throughout the growing season. The fruit sample was harvested from middle crown at 09:00–10:00 h from 10 to 120 DAFB on sunny days. Three fruit from three individual trees were pooled and subsequently treated as one biological replicate. Peel was removed from fruit sample tissue using a fruit paring knife and immediately frozen in liquid nitrogen and stored at −80°C until further analysis. Approximately 10 fruit randomly chosen from 12 trees were used to analyze fresh weight and diameter during developing stage.

### Sorbitol limitation

Approximately three fruit per m^2^ leaf area is the optimum source:sink (leaf:fruit) ratio for ‘Gala’ apple fruit to optimize fruit growth (McArtney et al., 1996). To limit sorbitol content, the leaves, which act as the source for ‘Gala’ apple fruit, were heavily thinned at 70 DAFB. The source:sink ratio of the thinned trees was 10:1 (10 fruit per m^2^ leaf area). The source-limited fruit were collected on days zero, one, three, six, seven, and nine after thinning. Three fruit from three individual trees were pooled and subsequently treated as one biological replicate.

### Feeding fruit with exogenous sugars

The source-limited (sorbitol-limited) apples were treated for five days and used for infiltration. The recipient apple was punctured according to the method of Xie et al. (2012) with some modifications. The infiltrations were performed using a 2-ml hypodermic needle (Kang Zheng, Jiangsu, China) coupled with a syringe ﬁlter (Pall Gelman Corp., Ann Arbor, MI, USA). Approximately 500 µl of sugars (50 mM sucrose and 100 mM hexose sugars) were injected into the chamber (space of the apple core) from the bottom of apple fruit. After injection, the pinhole was rubbed with 75% (v/v) alcohol and covered with 75% (v/v) alcohol-soaked foam using surgical tape. Tissue samples 1 cm in width were collected from the area surrounding the core of the injected fruit at 2 and 4 days after treatment. Three treated fruit from three individual trees were pooled and subsequently treated as one biological replicate.

### Metabolite analysis

Soluble sugars (sucrose, glucose, fructose, and sorbitol) were extracted and determined according to Liang et al. (2011) with some modifications. Frozen tissue (1.0 g) was homogenized with 3 ml of 80% (v/v) methanol and incubated at 80°C for 30 min followed by centrifugation for 10 min at 12,000 ***g***. Each pellet was then extracted twice with an additional 2 ml of 80% (v/v) methanol. The collected supernatants were pooled and evaporated at 55°C. The residue was reconstituted in 2 ml ultra-pure water (Milli-Q Academic A10^®^, Millipore, USA). The resulting solution was filtered using a 0.22-μm polypropylene syringe ﬁlter (Pall Gelman Corp., Ann Arbor, MI, USA), and the filtrate was stored at −20°C until further analysis using the HPLC-RID system. The HPLC-RID system is comprised of a Series 1260 Infinity^®^ HPLC (Agilent Technologies, CA, USA) with a1260^®^ Refractive Index Detector (Agilent Technologies, CA, USA). The HPLC-RID was equipped with a NH2P-50 4E^®^ column (4.6×250 mm) and a NH2P-50 4A^®^ guard column (Shodex Asahipak, Tokyo, Japan) using an isocratic program consisting of 75% (v/v) acetonitrile at a flow rate of 1.0 ml min^−1^. All separations were maintained at 40°C, and the injection volume of each sample was 10.0 μl.

Tre6P and phosphorylated intermediates were assayed according to Lunn et al. (2006). Trehalose was quantified as described by Holovati and Acker (2007). Starch was analyzed according to Fredeen et al. (1988) with slight modifications. The starch-containing pellet was dried at 55°C and subsequently dissolved in 3 ml H_2_O after boiling for 2 h. 3 ml of 0.1 N acetate buffer (pH 4.5) and 0.4 ml of amyloglucosidase (EC 3.2.1.3) were added to the starch-containing fraction. The mixture was subsequently incubated at 55°C for 24 h. After incubation, the tubes were spun at 9000 ***g*** for 10 min and the supernatant was retained for further analysis. The glucose content was analyzed using a 1260 Infinity^®^ HPLC-RID (Agilent Technologies, CA, USA). The glucose values were multiplied by 0.9 for conversion to starch.

### Statistical analysis

Statistical analysis was performed using SPSS 13.0. The data were compared using Student's *t*-test. Differences at *P*<0.05 were considered significant.
